# Effects of EHD2 interference on migration of esophageal squamous cell carcinoma

**DOI:** 10.1007/s12032-012-0396-4

**Published:** 2013-01-25

**Authors:** Mei Li, Xiaojing Yang, Jianguo Zhang, Hui Shi, Qinglei Hang, Xianting Huang, Guoliang Liu, Junya Zhu, Song He, Huijie Wang

**Affiliations:** 1 Department of Medical Oncology, Fudan University Shanghai Cancer Center, No 270 Dongan Road, Shanghai, 200032 People’s Republic of China; 2 Department of Oncology, Shanghai Medical College, Fudan University, No 270 Dongan Road, Shanghai, 200032 People’s Republic of China; 3 Department of Pathology, Nantong University Cancer Hospital, Nantong, Jiangsu 226001 People’s Republic of China; 4 Department of Immunology, Medical College, Nantong University, Nantong, Jiangsu 226001 People’s Republic of China

**Keywords:** EHD2, Esophageal squamous cell carcinoma, Migration, Prognosis

## Abstract

C-Terminal EH domain-containing protein 2 (EHD2) of the EHD family is associated with plasma membrane. We investigated the expression of EHD2 in human esophageal squamous cell carcinoma (ESCC) and the EHD2 expression to study the therapeutic effect of chemotherapy drugs. Western blot and immunohistochemistry were used to measure the expression of EHD2 protein in ESCC and adjacent normal tissue in 98 patients. EHD2 protein level was reduced in ESCC tissues in comparison with adjacent normal tissues. Under-expression of EHD2 increased the motility property of ESCC cell TE1 in vitro by wound-healing assays and transwell migration assays, and it was concurrent with the decreased expression of epithelial marker E-cadherin. Under-expression of EHD2 in TE1 can cause resistance to cisplatin. Our results suggested that EHD2 low expression is involved in the pathogenesis of ESCC, and it might be a favorable independent poor prognostic parameter for ESCC.

## Introduction

Esophageal squamous cell carcinoma (ESCC) is one of the most common clinical malignancies [1]. There is an exceedingly high incidence of ESCC in Asian countries, especially in north and central China [2]. ESCC is a highly aggressive disease, and the 5-year survival rate is approximately 15 % [3]. Tumor metastasis plays an important role in the development of malignant tumors. Hence, identifying functional metastasis genes and their molecular mechanisms underlying the metastatic process remains a top priority in the cancer research field.

Although 90 % of cancer deaths are caused by metastasis [4], the mechanism of cancer metastasis remains poorly defined and knowledge of this process will provide great promise for cancer therapy. Studies have shown that the actin cytoskeleton plays an essential role in numerous aspects of cell biology such as cell adhesion, cell morphology, cytokinesis, and especially in migration [5]. EHD2 (C-Terminal EH domain-containing protein 2) is a plasma membrane-associated member of the EHD family, which regulates internalization and is related to actin cytoskeleton. EHD2 was linked to the plasma membrane through actin filaments [6]. There have been reports about EH protein found in tumor, such as acute myeloid leukemias [7], but there is not any in-depth study of its mechanism. EHD2 plays a role in membrane reorganization in response to nucleotide hydrolysis which binds to liposomes and deforms them into tubules. Under-expression of EHD2 has been reported in malignant serous ovarian cancer samples as compared with primary cultures of normal ovarian surface epithelial samples [8]. EHD2 has also been implicated as a tumor suppressor gene candidate mapping to a 1.6 Mb 19q region of deletion in glioma tumors [9].

Abnormal expression of EHD2 is closely related to metastasis of carcinoma. We examined the EHD2 expression in ESCC and found that EHD2 played an important role in ESCC metastasis. Interference of EHD2 led to esophageal squamous cell carcinoma cells TE1 migration significantly. Cisplatin made the TE1 cells interfered of EHD2 which were harder to apoptosis than the normal TE1 cells. We can see that EHD2 has a low expression which leads to metastasis of ESCC.

## Materials and methods

### Tissue specimens and immunohistochemical analyses

A total of 98 ESCC specimens were retrieved from the archival files of the Department of Pathology, affiliated Hospital of Nantong University from 2005 to 2011. All human tissues were collected using protocols approved by the Ethics Committee of Nantong University Cancer Hospital. None of the patients was treated with such preoperative therapies as radiation, chemotherapy, or immunotherapy. Resected specimens were classified according to the International Union against Cancer TNM classification system [10]. The clinical data were collected after patients gave informed consent. The study population consisted of 71 males and 27 females, and the age ranged from 31 to 85 years.

Serial sections measuring 5 μm thick were mounted on glass slides coated with 10 % polylysine. Sections were dewaxed in xylene and rehydrated in graded ethanols. Immunoreactivity was enhanced by high temperature and pressure and incubating the tissue sections for 3 min in 0.1 mol/L citrate buffer. The following panel of antibodies was used: (1) EHD2 (1:100, Santa Cruz Biotechnology) and (2) E-cadherin (1:1,000, Santa Cruz Biotechnology). Immunostaining was performed using the avidin–biotin–peroxidase complex method, and antigen–antibody reactions were visualized with chromogen diaminobenzidine. Similar tissue sections immunostained with nonspecific immunoglobulin G were used as negative controls. Five high-power fields were randomly chosen, and at least 300 cells were counted per field. Expression score was determined by staining intensity and immunoreactive cell percentage. Tissues with no staining were rated as 0, with a faint staining or moderate to strong staining in ≤25 % of cells as 1, with moderate staining or strong staining in 25–50 % of cells as 2, strong staining in ≥50 % of cells as 3. For statistical analysis, <2 were counted as low expression, while ≥2 were counted as overexpression.

### Western blot analysis

Western blot experiments were used to measure certain proteins. Briefly, the cells were lysed in lysis buffer (120 mM Tris (pH 7.4), 135 mM NaCl, 1 mM EDTA, 1 % NP40, 0.1 % SDS, 1 mM Na_3_VO_4_, 1 mM aprotinin, and 1 mM PMSF). An equivalent amount of protein from each sample was electrophoresed by 12 % sodium dodecyl sulfate–polyacrylamide gel electrophoresis (SDS–PAGE) and then transferred to a PVDF membrane. After blocking with phosphate-buffered saline (PBS) containing 5 % nonfat milk and 0.1 % Tween 20 overnight, the membrane was incubated with primary antibody at 4 °C overnight. After washing with PBS containing 0.1 % Tween 20 three times, each for 5 min, the membrane was then incubated with HRP-labeled secondary antibody for another 2 h at room temperature. The membrane was then developed using the ECL detection systems.

The antibodies used in this study included: anti-EHD2 (anti-rabbit, 1:500, Santa Cruz Biotechnology), anti-E-cadherin (anti-mouse, 1:1,000, Santa Cruz Biotechnology), and anti-GAPDH (anti-rabbit, 1:1,000, Sigma).

### Cell culture and transfection

Human ESCC cell lines TE1 were obtained from our laboratory. Cells were maintained in RPMI1640 (Invitrogen) supplemented with 10 % fetal bovine serum (Invitrogen), 100 U/mL penicillin and 100 μg/mL streptomycin, within a humidified atmosphere containing 5 % CO_2_ at 37 °C. Cell transfection was performed with SuperFectin according to the manufacturer’s instructions.

### Wound-healing assay

TE1 cells were seeded on 6-well plates at a density of 5 × 10^5^ cells/well. After the cells reached sub-confluence, the monolayer cells were wounded by scraping off the cells and then grown in medium for 48 h. The migrated distance of cells was monitored and imaged under a microscope. The distances of cell migration were calculated by subtracting the distance between the lesion edges at 48 h from the distance measured at 0 h. The relative migrating distance of cells is measured by the distance of cell migration/the distance measured at 0 h.

### Transwell assay

Cell migration was determined using a Transwell (Corning, NY, USA) with a pore size of 0.8 μm. 1 × 10^5^ cells were seeded in serum-free medium in the upper chamber (normal chamber for migration assay and matrigel-coated chamber for invasion assay), while medium containing 10 % FBS in the lower chamber. After incubating for 8 h at 37 °C, cells in the upper chamber were carefully removed with a cotton swab and the cells that had traversed to reverse face of the membrane were fixed in methanol, stained with Giemsa, and counted [11].

### Cell counting kit-8 assay

After treatment according to the protocols, cells were seeded at 3 × 10^4^/well in 100 μL medium in 96-well plates and incubated overnight to allow cell adherence. Cells were then exposed to various concentrations of cisplatin for 48 h, monosodium salt (Dojindo, Kumamoto, Japan) was added to each well, and the culture plate was incubated at 37 °C for 1 h. Absorbance was measured at 450 nm.

### Plasmid constructs

The siRNA species purchased from GenePharma were designed to target the following cDNA sequences: scrambled, 5′-CAGTCGCGTTTGCGACTGG-3′; EHD2-siRNA, 5′-AAGAA AGAGATGCCCACGGTGTT-3′.

### Statistical analysis

Statistical analysis was performed using the PASW statistics 18 software package. The association between EHD2 expression and clinicopathological features was analyzed using the χ^2^ test. As the data were not normally distributed, EHD2 and E-cadherin expressions were studied using the Spearman rank correlation test. For the analysis of survival data, Kaplan–Meier curves were constructed and the log-rank test was performed. Multivariate analysis was performed using Cox’s proportional hazards model, with *P* < 0.05 considered statistically significant. The results are expressed as the mean ± SE.

## Results

### EHD2 expression and its correlation with clinicopathologic variables in ESCC

**Fig. 1:**
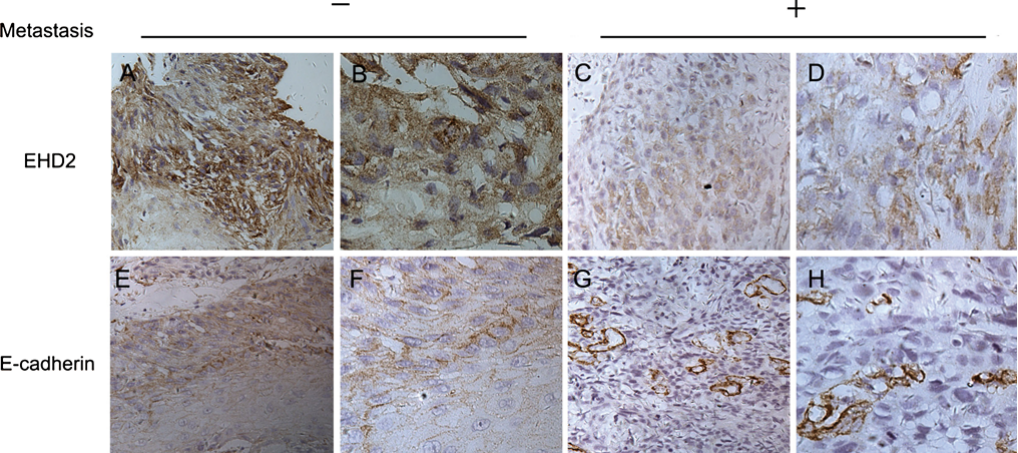
Immunohistochemical staining reveals EHD2 and E-cadherin expression in paraffin-embedded ESCC tissues. **a**, **b**, **e**, **f** Cancer tissues with no lymph node metastasis showed high EHD2 and E-cadherin expression (×200 and ×400). **c**, **d**, **g**, **h** Cancer tissues with lymph nodes metastases showed low EHD2 and E-cadherin expression (×200 and ×400). Details of the experiments are given in “Materials and methods”

We used immunohistochemical staining to detect the expression of EHD2 and E-cadherin in 98 ESCC samples. As shown in Fig. 1, increased membranous and expression of EHD2 existed in the sample tissues with no lymph node metastasis. The typical case showed that low expression of EHD2 was correlated with low E-cadherin in the same esophageal cancer specimen. The results of 98 ESCC samples by immunohistochemical analyses are in Table 1, and the patients were divided into two groups: high EHD2 expressers (score ≥ 2) and low EHD2 expressers (score < 2). EHD2 expression correlates significantly with tumor metastasis (*P* < 0.001) and histological grade (*P* = 0.001) but there was no relationship between EHD2 expression and other prognostic factors like tumor size and invasion.

**Table 1:** EHD2 expression and clinicopathological parameters in 98 esophageal cancer specimens

Parameters	Total	EHD2		*P*
		Score < 2, *n* (%)	Score ≥ 2, *n* (%)	
Age (years)				
<60	37	17(45.9)	20(54.1)	0.225
≥60	61	22(36.1)	39(63.9)	
Gender				
Male	71	30(42.3)	41(57.7)	0.285
Female	27	9(33.3)	18(66.7)	
Tumor grade				
I	17	1(5.9)	16(94.1)	0.001*
II	47	19(40.4)	28(59.6)	
III	34	19(55.9)	15(44.1)	
Metastasis				
Presence	65	13(20.0)	52(80.0)	0.000*
Absence	33	26(78.8)	7(21.2)	
Tumor size (cm)				
<5	75	28(37.3)	47(62.7)	0.255
≥5	23	11(47.8)	12(52.2)	
Tumor invasion (T)				
T1	11	4(36.4)	7(63.6)	0.423
T2	15	8(53.3)	7(46.7)	
T3	25	7(28.0)	18(72)	
T4	47	20(42.6)	27(57.4)	

Statistical analyses were performed by Pearson χ^2^ test

* *P* < 0.05 was considered significant

**Fig. 2:**
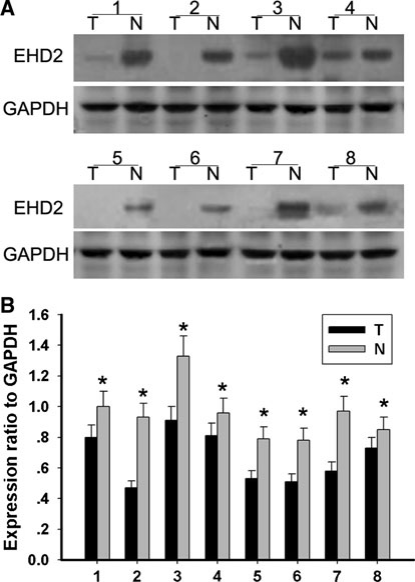
Expression of EHD2 in human ESCC. **a** Expression of EHD2 in eight representative paired samples of esophageal tumor tissues (T) and adjacent normal tissues (N). **b** The bar chart demonstrates the ratio of EHD2 protein to GAPDH for the above by densitometry. The data are mean ± SEM (*P < 0.01, compared with adjacent tumor tissues)

To confirm the specificity of the immunohistochemical results, Western bolt analysis was carried out in eight esophageal tumor tissues, in which freshly frozen materials were available. The expression of EHD2 was examined for Western blot analysis, which showed accordant result with immunohistochemistry. The example of Western blot analysis is shown in Fig. 2. Lower expression of EHD2 was observed in malignant esophageal tumors (T) than in adjacent normal tissues (N). The amount of GAPDH, a housekeeping protein, was demonstrated to be rather constant among the samples.

### Correlation between expression of EHD2 and survival rates in patients with ESCC

**Fig. 3:**
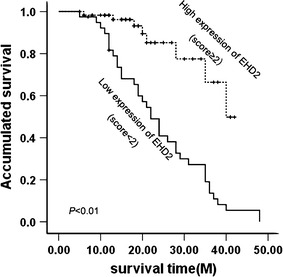
Cumulative survival curves according to EHD2 expression. On the basis of score of EHD2, patients were divided into high EHD2 expressers (score ≥ 2) and low EHD2 expressers (score < 2). Patients in the low-expression EHD2 group had significantly shorter overall survival

Survival analysis was restricted to 98 patients with available complete follow-up data and results of EHD2 expressions. By using the Kaplan–Meier analysis, patients with low expression of EHD2 are significantly associated with short overall survival (*P* < 0.01; Fig. 3). Multivariate analysis using the Cox’s proportional hazards model showed that EHD2 protein is an independent prognostic indicator for patients’ overall survival (*P* = 0.000; Table 2).

**Table 2:** Contribution of various potential prognostic factors to survival by Cox regression analysis in 98 specimens

	Relative ratio	95 % confidence interval	*P*
Age (years)	1.457	0.783–2.712	0.235
Gender	0.825	0.423–1.609	0.572
Tumor size	0.994	0.509–1.944	0.986
Metastasis	2.271	1.247–4.137	0.007*
Tumor invasion	1.024	0.761–1.378	0.876
Tumor grade	1.908	1.286–3.050	0.002*
EHD2	0.205	0.095–0.445	0.000*

Statistical analyses were performed by Cox test

* *P* < 0.05 was considered significant

### Interference of EHD2 expression inhibits the migration of TE1 cells

**Fig. 4:**
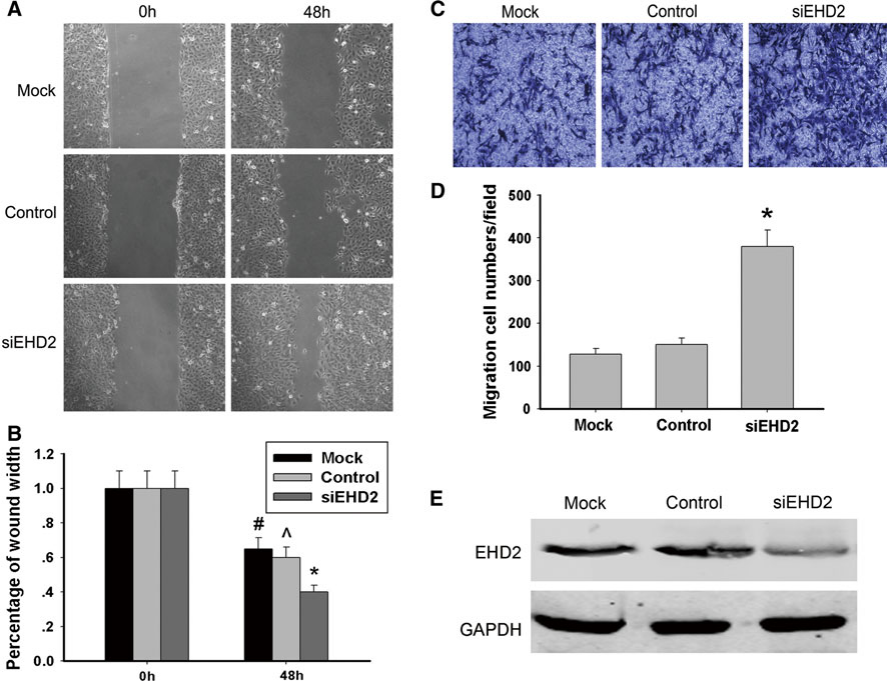
Under-expression of EHD2 facilitates the migration and invasion of TE1 cells. Migration and invasion of cells treated with siEHD2 (or negative control) were analyzed at 48 h post-infection. **a** Wound-healing assay. Photographs represented the cells migrated into the wounded area and **b** histogram showed the relative migration distance of cells. The data are mean ± SEM (*n* = 3, ^#,^,^*P < 0.01, compared with 0 h). **c** Transwell assay. Photographs represented the cells travelled through the micropore membrane and **d** histogram showed the percentage of migrant cells. The data are mean ± SEM (*n* = 3, *P < 0.01, compared with Mock). **e** EHD2 interference efficiency was certified by Western blot. Data represent mean ± SEM from four independent experiments; *P* < 0.01 by *t* test

Tumor metastasis is the leading cause of low survival rate of ESCC patients [12]. To investigate the role of EHD2 in ESCC metastasis, we detected the migrant capacity of ESCC TE1 cell which was interfered or non-interfered with siEHD2. Through transwell and wound-healing assay, we found that the percentage of cells that travelled through the micropore membrane was significantly increased (Fig. 4c), and the relative migrating distance of cells was significantly longer (Fig. 4a) in TE1 siEHD2 cells as compared with the non-interfered cells. SiRNA interference plasmid was used in the interference expression of EHD2, and Western blot was used for result verification (Fig. 4e). These results indicate that low expression of EHD2 inhibits the migration of ESCC TE1 cells.

### EHD2 expression is closely related to chemotherapy drugs on the treatment for ESCC

**Fig. 5:**
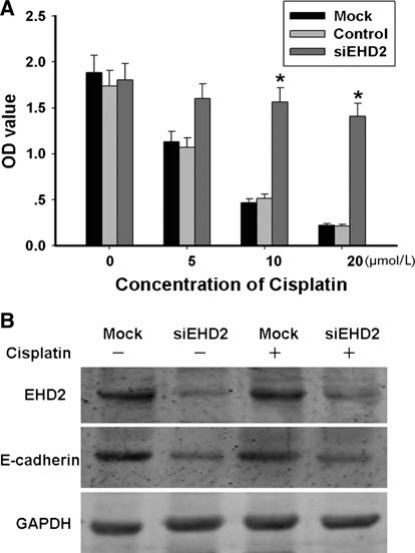
Interference of EHD2 affected TE1 cells proliferation and was insensitive to chemotherapy drugs. **a** Treat TE1 cells with cisplatin for 48 h at 5, 10, 20 μmol/L, respectively, after interference of EHD2 for 48 h. The data are mean ± SEM (*n* = 3, *P < 0.01, compared with Mock). **b** Western blot analyses proliferation of TE1 cells when interference of EHD2 and with or without treatment of ciaplatin

We speculated that under-expression of EHD2 reduced chemosensitivity in tumor cells. Therefore, we tested whether siEHD2 affected the response to cisplatin treatment in ESCC TE1 cells. TE1 cells were transfected with siEHD2 plasmid and were treated with various concentrations of cisplatin for 48 h. The result of the Cell Counting Kit-8 (CCK8) assay indicated that the transfection of siEHD2 significantly increased cell viability compared with mock transfection. At concentrations higher than 10 μmol/L, the survival rate of the cells that were transfected with siEHD2 was significantly higher than that of the cells that were transfected with mock and negative control (Fig. 5a). As stated above, the under-expression of EHD2 was insensitive to cisplatin in TE1 cells. Epithelial marker E-cadherin was met in these mock and siEHD2 transfected TE1 cells whether treated with cisplatin or not (Fig. 5b). Low expression of EHD2 being found in TE1 cells, the expression of E-cadherin was also decreased at the same time. In conclusion, EHD2 is closely related to the metastasis of TE1 cells. EHD2 can be used as one of the prognostic factors of ESCC.

## Discussion

Generally, our results demonstrated that EHD2 expression in the cancer with lymph nodes metastases decreased significantly, which indicated that under-expression of EHD2 had a potential relation with the severity of malignancy of ESCC. EHD2 was bound up with indicators of metastases, such as E-cadherin. There was a statistically close relationship between survival rate and EHD2 expression in ESCC. These findings are in consistent with the observations in ovarian serous carcinomas previously reported [13]. And we discovered that under-expression of EHD2 was closely related with metastasis of ESCC. As a result, low expression of EHD2 correlated significantly with a poor prognosis. The survival rate of patients with low expression of EHD2 was lower than that of other patients, which suggested that the degree of expression of EHD2 might have an effect on the survival rate.

The nature of the most malignancy and primary cause of cancer treatment failure are invasion and metastasis [14]. It was reported that there were linkages of EHD2 to the actin cytoskeleton which provides strong evidence that EHD2 functions at some point in endocytosis at which F-actin facilitates the process [6]. The study found that the expression level of EHD2 was significantly lower than that of normal tissue, and EHD2 expression gradually decreased with pathological grading increased, which indicating EHD2 might be a cancer suppressor gene of ESCC.

Further experiments presented here suggested TE1 cells interference of EHD2 had a higher migration speed than that of normal which was verified by wound healing and transwell assay. After treated with different concentrations of cisplatin, siEHD2 TE1 cells were not easier to apoptosis than mock and negative control cells.

After interference of EHD2, the ESCC cell line TE1 accelerated cell growth speed which further supported a potential assumption that EHD2 was a tumor suppressor of ESCC. EHDs (EH (Eps15 homology)-domain-containing proteins) participate in different stages of endocytosis. EHD2 is a plasma membrane-associated EHD which regulates trafficking from the plasma membrane and recycling [15]. The EH domain of plant EHDs bears 32 % homology to the mammalian EHDs EH domain but also regulates endocytosis [16]. Our data showed that cells migration increased with EHD2 under-expression which might foreshadowed cells morphology changed by transcytosis cytoskeletal molecules. This remains to be verified by follow-up experiments. Now, with the research of the theory and mechanism of intracellular signal transduction going deeper, people start to see signaling molecules of the receptor and downstream signal transduction pathway as a target for therapeutic intervention strategy [17–19]. Some achievements have been obtained on the basis of tumor therapeutic drug development while new mechanism and new theory still need to be developed and practiced [20]. This research focuses on the exploration of the role that membrane transport proteins play in tumor biology. These proteins play an important role on various membrane proteins and the various types of receptor endocytosis transporter regulation. Thus, such proteins may impact in the various stages of tumorigenesis [21, 22]. There have been reports about that endocytic proteins in the regulation of nuclear signaling, transcription, and tumorigenesis [23], but further study on molecular mechanisms remains to be scheduled.

In summary, the results of this study and related findings suggest that EHD2 is likely to be a new ESCC suppressor gene. Disorder of EHD2 expression in tumor tissue may cause esophageal squamous cell structure to change and obtain the migration ability. Further research of the mechanism how EHD2 potential regulates the cytoskeleton molecular will broaden the understanding of the tumor moleculars and cell biology, which may provide a new target and new ideas for the diagnosis and treatment for ESCC.
